# Supplementary material for: Heat adaptation of phage T7 under an extended genetic code
Source: Virus Evol. 2021 Dec 1;7(2):veab100. doi: 10.1093/ve/veab100 (PMC8923235; doi:10.1093/ve/veab100)
Supplement: veab100_Supp [file veab100_supp.zip › Sequencing_Analysis.zip!/Sequencing_Analysis/S1/evidence/JC_SIDE_2_47.html]

BRESEQ :: Evidence


| New junction evidence | | | | | | | | | | | |
| --- | --- | --- | --- | --- | --- | --- | --- | --- | --- | --- | --- |
|  | | seq id | position | reads (cov) | reads (cov) | score | skew | freq | annotation | gene | product |
| \* | ? | NC\_001604.1 | = 1256 | 0 (0.000) | 154 (0.040) | 67/574 | 22.6 | 99.7% | intergenic (–/–) | *–/–* | –/– |
|  | ? | NC\_001604.1 | 2737 = | 1 (0.000) |  |  |  |  | intergenic (–/–) | *–/–* | –/– |
| Rejected: Coverage evenness skew score above cutoff. | | | | | | | | | | | |

`CATTCAGCATCGACCCTGAGGAACTCATCAAGGAAGTCGAGGAAGTCGCACGACAGAAAGAAATTGACCGCGCTAAGGCCCGTAAAGAACGTCACGAGGGGCGCTTAGAGGCACGCAGATTCAAACGTCGCAACCGCAA‑GGCACGTAAAGCACACAAAGCTAAGCGCGAAAGAATGCTTGCTGCGTGGCGATGGGCTGAACGTCAAGAACGGCGTAACCATGAGGTAGCTGTAGATGTACTAGGAAGAACCAATAACGCTATGCTCTGGGTCAACATGTTCTCTGGGGACTTTAAGGCGCTTGAGGAACGAATCGCGCTGCACTGGCGTAATGCTG  >  NC_001604.1/2694‑3029`  

                                           `|`                                                                                                                                                                                                                                                                                                       
`acgcagaagacttgctcaatgaatacttggaggaagtcgaggaagtcgCACGACAGAAAGAAATTGACCGCGCTACGGCCCGTAAAGAACGTCACGAGGGGCGCTTAGAGGCACGCAGATTCAAACGTCGCAACCGCAA‑GGCACGTAAAGCACACAAAGCTAAGCGCGAAAGAATGCTTGCTGCGTGGCGATGGGCTGAACGTCAAGAACGGCGTAACCATGAGGTAGCTGTAGATGTACTAGGAAGAACCAATAACGCTATGCTCTGGGTCAACATGTTCTCTGGGGCCTTTAAGGCGCt                                     >  1:96261/31‑301 (MQ=255)`  
`acgcagaagacttgctcaatgaatacttggaggaagtcgaggaagtcgCACGACAGAAAGAAATTGACCGCGCTAAGGCCTGTAAAGAACGTCACGAGGGGCGCTTAGAGGCACGCAGATTCAAACGTCGCAACCGCAA‑GGCACGTAAAGCACACAAAGCTAAGCGCGAAAGAATGCTTGCTGCGTGGCGATGGGCTGGACGTCAAGAACGGCGTAACCATGAGGTAGCTGTAGCTGTACTAGGAAGAACAAATAACGCTATGCTCTGGGTCAACATGTTCTCTGGGGACTTTAAGGCGCt                                     >  1:653417/31‑301 (MQ=255)`  
`acgcagaagacttgctcaatgaatacttggaggaagtcgaggaagtcgCACGACAGAAAGAAATTGACCGCGCTAAGGCCCGTAAAGAACGTCACGAGGGGCGCTTAGAGGCACGCAGATTCAAACGTCGCAACCGCAA‑GGCACGTAAAGCACACAAAGCTAAGCGCGAAAGAATGCTTGCTGCGTGGCGATGGGCTGAACGTCAAGAACGGCGTAACCATGAGGTAGCTGTAGATGTACTAGGAAGAACCAATAACGCTATGCTCTGGGTCAACATGTTCTCTGGGGACTTTAAGGCGCt                                     >  1:20312/31‑301 (MQ=255)`  
  `gcagaagacttgctcaatgaatacttggaggaagtcgaggaagtcgCACGACAGAAAGAAATTGACCGCGCTAAGGCCCGTAAAGAACGTCACGAGGGGCGCTTAGAGGCACGCAGATTCAAACGTCGCAACCGCAA‑GGCACGTAAAGCACACAAAGCTAAGCGCGAAAGAATGCTTGCTGCGTGGCGATGGGCTGAACGTCAAGAACGGCGTAACCATGAGGTAGCTGTAGATGTCCTAGGAAGAACCAATAACGCTATGCTCTGGGTCAACATGTTCTCTGGGGACTTTAAGGCGCTTg                                   >  1:156091/29‑301 (MQ=255)`  
  `gcagaagacttgctcaatgaatacttggaggaagtcgaggaagtcgCACGACAGAAAGAAATTGACCGCGCTAAGGCCCGTAAAGAACGTCACGAGGGGCGCTTAGAGGCACGCAGATTCAAACGTCGCAACCGCAA‑GGCACGTAAAGCACACAAAGCTAAGCGCGAAAGAATGCTTGCTGCGTGGCGATGGGCTGAACGTCAAGAACGGCGTAACCATGAGGTAGCTGTAGATGTACTAGGAAGAACCAATAACGCTATGCTCTGGGTCAACATGTTCTCTGGGGACTTTAAGGCGCTTg                                   >  1:654547/29‑301 (MQ=255)`  
   `cagaagacttgctcaatgaatacttggaggaagtcgaggaagtcgCACGACAGAAAGAAATTGACCGCGCTAAGGCCCGTAAAGAACGTCACGAGGGGCGCTTAGAGGCACGCAGATTCAAACGTCGCAACCGCAA‑GGCACGTAAAGCACACAAAGCTAAGCGCGAAAGAATGCTTGCTGCGTGGCGATGGGCTGAACGTCAAGCACGGCGTAACCATGAGGTAGCTGTAGATGTACTAGGAAGAACCAATAACGCTATGCTCTGGGTCAACATGTTCTCTGGGGACTTTAAGGCGCTTGa                                  >  1:527652/28‑301 (MQ=255)`  
   `cagaagacttgctcaatgaatacttggaggaagtcgaggaagtcgCACGACAGAAAGAAATTGACCGCGCTAAGGCCCGTAAAGAACGTCACGAGGGGCGCTTAGAGGCACGCAGATTCAAACGTCGCAACCGCAA‑GGCACGTAAAGCACACAAAGCTAAGCGCGAAAGAATGCTTGCTGCGTGGCGATGGGCTGAACGTCAAGAACGGCGTAACCATGAGGTAGCTGTAGATGTACTAGGAAGAACCAATAACGCTATGCTCTGGGTCAACATGTTCTCTGGGGACTTTAAGGCGCTTGa                                  >  1:316926/28‑301 (MQ=255)`  
     `gaagacttgctcaatgaatacttggaggaagtcgaggaagtcgCACGACAGAAAGAAATTGACCGCGCTAAGGCCCGTAAAGAACGTCACGAGGGGCGCTTAGAGGCACGCAGATTCAAACGTCGCAACCGCAA‑GGCACGTAAAGCACACAAAGCTAAGCGCGAAAGAATGCTTGCTGCGTGGCGATGGGCTGAACGTCAAGAACGGCGTAACCATGAGGTAGCTGTAGATGTACTAGGAAGAACCAATAACGCTATGCTCTGGGTCAACATGTTCTCTGGGGACTTTAAGGCGCTTGAgg                                <  1:548947/276‑1 (MQ=255)`  
     `gaagacttgctcaatgaatacttggaggaagtcgaggaagtcgCACGACAGAAAGAAATTGACCGCGCTAAGGCCCGTAAAGAACGTCACGAGGGGCGCTTAGAGGCACGCAGATTCAAACGTCGCAACCGCAA‑GGCACGTAAAGCACACAAAGCTAAGCGCGAAAGAATGCTTGCTGCGTGGCGATGGGCTGAACGTCAAGAAAGGCGTAACCATGAGGTAGCTTTAGATGTACTAGGCAGAACCAATAACGCTATGCTCTGGGTCAACAGGTTCTTTGGGGGCTTTAAGGGGTCTGAgg                                >  1:640508/26‑301 (MQ=255)`  
     `aaagacttgctcaatgaatacttggaggaagtcgaggaagtcgCACGACAGAAAGAAATGGACCGCGCTAAGGCCCGTAAAGAACGTCACGAGGGGCGCTTAGAGGCACGCAGATTCAAACGTCGCAACCGCAA‑GGCACGTAAAGCACACAAAGCTAAGCGCGAAAGAATGCTTGCTGCGTGGCGATGGGCTGAACGTCAAGAACGGCGTAACCATGAGGTAGCTGTAGATGTACTAGGAAGAACCAATAACGCTATGCTCTGGGTCAACATGTTCTCTGGGGACTTTAAGGCGCTTGAgg                                <  1:131022/276‑1 (MQ=255)`  
       `agagttgctcaatgaatacttggaggaagtcgaggaagtcgCACGACAGAAAGAAATTGACCGCGCTAAGGCCCGTAAAGAACGTCACGAGGGGCGCTTAGAGGCACGCAGATTCAAACTTCGCAACCGCCA‑GTCACGTAAAGCCCACAAAGCTAAGCTCGAAAGAATGCTTGCTGCGTGGCGATGGGCTGAACGTCCAGAACGGCGTAACCATGAGGTAACTTTAGATGTACTAGGAAGAACCAATAACGCTATGCTCTGGGTCAACATGTTCTCTGGGTACTTTAAGGCGCTTCAGTaa                              >  1:110656/24‑301 (MQ=255)`  
       `agaattgctcaatgaatacttggaggaagtcgaggaagtcgCACGACAGAAAGAAAGTGACCGCGCTAAGGCCCGTAAAGAACGTCACGAGGGGCGCTTAGAGGCACGCAGATTCAAACGTCGCAACCGCAA‑GGCACGTACAGCACACAAAGCTAAGCGCGAAAGAATGCTTGCGGCGTGGCGATGGGCTGAACGTCAAGAACGGCGTAACCATGAGGTAGCTGTAGATGTACTAGGAAGAACCAATAACGCTATGCTCTGGGTCAACATGTTCTCTGGGGACTTTAAGGCGCTTGAGGaa                              <  1:565121/278‑1 (MQ=255)`  
       `acgagacagacatgaatacttggaggaagtcgaggaagtcgCACGACAGAAAGAACTTGACCGCGCTAAGGCCCGTAAAGAACGTCACGAGGGGCGCTTAGAGGCACGCAGATTCAAACGTCGCAACCGCAA‑GGCACGTAAAGCACACAAAGCTAAGCGCGAAAGAATGCTTGCTGCGTTGCGATGGGCTGAACGTCAAGAACGGCGTAACCATGAGGTAGCTGTAGATGTACTATGAAGAACCAATAACGCTATGCTCTGGGTCAACATGTTCTCTGGGGACTTTAAGGCGCTTGAGGaa                              <  1:617435/278‑1 (MQ=255)`  
       `aagagacagcaatgaatacttggaggaagtcgaggaagtcgCACGACAGAAAGAAATTGACCGCGCTAAGGCCCGTAAAGAACGTCACGAGGGGCGCTTAGAGGCACGCAGATTCAAACGTCGCAACCGCAA‑GGCACGTAAAGCACACAAAGCTAAGCGCGAAAGAATGCTTGCTGCGTTGCGATGGGCTGAACGTCAAGAACGGCGTAACCATGAGGTAGCTGTAGATGTACTATGAAGAACCAATAACGCTATGCTCTGGGTCAACATGTTCTCTGGGGACTTTAAGGCGCTTGAGGaa                              <  1:617429/278‑1 (MQ=255)`  
        `gacttgctcaatgaatacttggaggaagtcgaggaagtcgCACGACAGAAAGAAATTGACCGCGCTAAGGCCCGTAAAGAACGTCACGAGGGGCGCTTAGAGGCACGCAGATTCAAACGTCGCAACCGCAA‑GGCACGTAAAGCACACAAAGCTAAGCGCGAAAGAATGCTTGCTGCGTGGCGATGGGCTGAACGTCAAGAACGGCGTAACCATGAGGTAGCTGTAGATGTACTAGGAAGAACCAATAACGCTATGCTCTGGGTCAACATTTTCTCTGGGGACTTTAAGTCGCTTGAGGAAc                             >  1:85137/23‑301 (MQ=255)`  
        `gacttgctcaatgaatacttggaggaagtcgaggaagtcgCACGACAGAAAGAAATTGACCGCGCTAAGGCCCGTAAAGAACGTCACGAGGGGCGCTTAGAGGCACGCAGATTCAAACGTCGCAACCGCAA‑GGCACGTAAAGCACACAAAGCTAAGCGCGAAAGAATGCTTGCTGCGTGGCGATGGGCTGAACGTCAAGAACGGCGTAACCATGAGGTAGCTGTAGATGTACTAGGAAGAACCAATAACGCTATGCTCTGGGTCAACATGTTCTCTGGGGACTTTAAGTCGCTTGAGGAAc                             >  1:85140/23‑301 (MQ=255)`  
          `tgtataagagacagactgggaggaagtcgaggaagtcgCACGACAGAAAGAAATTGACCGCGCTAAGGCCCGTAAAGAACGTCACGAGGGGCGCTTAGAGGCACGCAGATTCAAACGTCGCAACCGCAA‑GGCACGTAAAGCACACAAAGCTAAGCGCGAAAGAATGCTTGCTGCGTGGCGATGGGCTGAACGTCAAGAACGGCGTAACCATGAGGTAGCTGTAGATGTACTAGGAAGAACCAATAACGCTATGCTCTGGGTCAACATGTTCTCTGGGGACTTTAAGGCGCTTGAAGAACGa                           <  1:581270/281‑1 (MQ=255)`  
          `catgctaaatgaatacgtggaggaagtcgaggaagtcgCACGACAGAAAGAAATTGACCGCGCTAAGGCACGTAAAGAACGTCACGAGGGGCGCTTAGAGGCATGCAGATTCAAACGTCGCAACCGCAA‑GGCACGTAAAGCACACAAAGCTAAGCGCGAAAGAATGCTTGCTGCGTGGCGATGGGCTGAACGTCAAGAACGGCGTAACCATGAGGTAGCTGTAGATGTACTAGGAAGAACCAATAACGCTATGCTCTGGGTCAACATGTTCTCTGGGGACTTTAAGGCGCTTGAGGAACGa                           <  1:331246/281‑1 (MQ=255)`  
          `attgctcaattaatacttggaggaagtctaggaattcgACCGACAGAAAGAAATTGACCGCGCTAAGGCCCGTAAAGAACGTCACGAGGGGCGCTTAGAGGCACGCAGATTCAAACGTCGCAACCGCAA‑GGCACGTAAAGCACACAAAGCTAAGCGCGAAAGAATGCTTGCTGCGTGGCGATGGGCTGAACGTCAAGAACGGCGTAACCATGAGGTAGCTGTAGATGTACTAGGAAGAACCAATAACGCTATGCTCTGGGTCAACATGTTCTCTGGGGACTTTAAGGCGCTTGAGGAACGa                           <  1:331254/281‑1 (MQ=255)`  
            `acagcaatgaagactgggaggaaggcgaggaagtcgCACGACAGAAAGAAATTGACCGCGCTAAGGCCCGTAAAGAACGGCACGAGGGGCGCTTAGAGGCACGCAGATTAAAACGTCGCAACCGCAA‑GGCACGTAAAGCACACAAAGCTAAGCGCGAAAGAATGCTTGCTGCGTGGCGATGGGCTGAACGTCAAGAACGGCGTAACCATGAGGTAGCTGTAGATGTACTAGGAAGAACCAATAACGCTATGCTCTGGGTCAACATGTTCTCTGGGGACTTTAAGGCGCTTGAGGAACGAAt                         <  1:457098/283‑1 (MQ=255)`  
             `gctcaatgaatacttggaggaagtcgaggaagtcgCACGACAGAAAGAAATTGACCGCGCTAAGGCCCGTAAAGAACGTCACGAGGGGCGCTTATAGGCACGCAGATTCAAACGTCGCAACCGCAA‑GGCACGTAAAGCACACAAAGCTAAGCGCGAAAGAATGCTTGCTGCGTGGCGATGGGCTGAACGTCAAGAACGGCGTAACCATGAGGTAGCTGTAGATGTACTAGGAAGAACCAATAACGCTATGCTCTGGGTCAACATGTTCTCTGGGGACTTTCAGGCGCTTTAGGAACGAATc                        >  1:85720/18‑301 (MQ=255)`  
             `gctcaatgaatacttggaggaagtcgaggaagtcgCACGACAGAAAGAAATTGACCGCGCTAAGGCCCGTAAAGAACGTCACGAGGGGCGCTTAGAGGCACGCAGATTCAAACGTCGCAACCGCAA‑GGCACGTAAAGCACACAAAGCTAAGCGCGAAAGAATGCTTGCTGCGTGGCGATGGGCTGAACGTCAAGAACGGCGTAACCATGAGGTAGCTGTAGATGTACTAGGAAGAACCAATAACGCTATGCTCTGGGTCAACATGTTCTCTGGGGACTTTAAGGCGCTTGAGGAACGAATc                        <  1:176541/284‑1 (MQ=255)`  
             `gctcaatgaatacttggaggaagtcgaggaagtcgCACGACAGAAAGAAATTGACCGCGCTAAGGCCCGTAAAGAACGTCACGAGGGGCGCTTAGAGGCACGCAGATTCAAACGTCGCAACCGCAA‑GGCACGTAAAGCACACAAAGCTAAGCGCGAAAGAATGCCTGCTGCGTGGCGATGGGCTGAACGTCCAGAACGGCGTAACCATGAGGTAGCTGTAGATGTACTAGGAAGCACCAATAAAGCTATGCTCTGGGTCAACATGTTCTCTGGGGACTTTAAGGAGCTTGAGGACCGAATc                        >  1:338284/18‑301 (MQ=255)`  
             `gctcaatgaatacttggaggaagtcgaggaagtcgCACGACAGAAAGAAATTGACCGCGCTAAGGCCCGTAAAGAACGTCACGAGGGGCGCTTAGAGGCACGCAGATTCAAACGTCGCAACCGCAA‑GGCACGTAAAGCACACAAAGCTAAGCGCGAAAGAATGCCTGCTGCGTGGCGATGGGCTGAACGTCAAGAACGGCGTAACCATGAGGTAGCTGTAGATGTACTAGGAAGAACCAATAACGCTATGACCTGGGTCAACATGTTCTCTGGGGACTTTCAGGCGCTTGAGGAACGAATc                        >  1:338268/18‑301 (MQ=255)`  
             `gctcaatgaatacttggaggaagtcgaggaagtcgCACGACAGAAAGAAATTGACCGCGCTAAGGCCCGTAAAGAACGGCACGAGGGGCGCTTAGAGGCACTCAGATTCAAACGTCGCAACCGCAA‑GGCACGTAAAGCACACAAAGCTAAGCGCGAAAGAATGCTTGCTGCGTGGCGATGGGCTGAACGTCAAGAACGGCGTAACCATGAGGCAGCTGTAGATGTACTAGGAAGAACCAATAACGCTATGCTCTGGGTCAACATGTTCTCTGGGGACTTTAAGGCGCTTGAGGAACGaagc                        >  1:290290/18‑299 (MQ=255)`  
              `gaggacttgctcaagaaaaactcgtgggaggtctCACGCAAGAAAGAAATAGTCCGCGTTAACTCGCGTAAAGAACGTGACGAGGGGTGTGTAGAGGCGCGGAAATTCAAACGTCGCAACCGAGA‑GTAACTTAAAACAACCAAAGCTAAGCGCGCAAGAATGCTTGCGGCGTGGCTATGGGCTGAACCTAAAGAACGGCGTACTCATGAGGTAGCTGTAGATGTACTAGGAAGAACCAATATCCCGATGCTATGGGTCAACATGTTCTATGGGGACTTTAAGGCGCTTGAGGAACGAATcg                       <  1:645651/275‑1 (MQ=255)`  
              `ctcaatgaatacttggaagaagtcgaggaagtcgCAAGACAGAAAAGAATTGACCGCGCTAAGGCCCGTAAAGAACGTCACGAGGGGCGCTTAGAGGCACGCAGATTCAAACGTCGCAACCGCAA‑GGCACGTAAAGCACACAAAGCTAAGCGAGAAAGAATGCTTGCTGCGTGGCGATGGGCTGAACGTCAAGAACGGCGTAACCATGAGGTAGCTGTAGATGTACTAGGAAGAACCAATAACGCTATGCTCTGGGTCAACATGTTCTCTGGGGACTTTAAGGCGCTTGAGGAACGAATcg                       <  1:638719/283‑1 (MQ=255)`  
                `caatgaatacttggaggaagtcgaggaagtcgCACGACAGAAAGAACTTGACCGAGCTAAGGCCCGTAAAGAACGTCACGAGGGGCGCTTAGAGGCACGCAGATTCAAACGTCGCAACCGCAA‑GGCACGTAAAGCACACAAAGCTAAGCGCGAAAGAATGCTTGCTGCGTGGCGATGGGCTGAACGTCAAGAACGGCGTAACCATGAGGTAGCTGTAGATGTACTAGGAAGAACCAATAACGCTATGCTCTGGGTCAACATGTTCTCGTGGGACTTTCAGGTGCTTTAGGAACGAAAcgcg                     >  1:100281/15‑301 (MQ=255)`  
                `caatgaatacttggaggaagtcgaggaagtcgCACGACAGAAAGAAATTGACCGCGCTAAGGCCCGTAAAGAACGTCACGAGGGGCGCTTAGAGGCACGCAGATTCAAACGTCGCAACCGCAA‑GGCACGTAAAGCACCCAAAGCTAAGCGCGAAAGAATGCTTGCTGCGTGGCGATGGGCTGAACGTCAAGAACGTCGTAACCATGAGGTAGCTGTAGATGTACTAGGAAGAACCAATAACGCTATGCTCTGGGTCAACATGTTCTCTGGGGACTTTAAGGCGCTTTAGGAACGAATcgcg                     >  1:296904/15‑301 (MQ=255)`  
                `caatgaatacttggaggaagtcgaggaagtcgCACGACAGAAAGAAATTGACCGCGCTAAGGCCCGTAAAGAACGTCACGAGGGGCGCTTAGAGGCACGCAGATTCAAACGTCGCAACCGCAA‑GGCACGTAAAGCACACAAAGCTAAGCGCGAAAGAATGCTTGCTGCGTGGCGATGGGCTGAACGTCAAGAACGGCGTAACCATGAGGTAGCTGTAGATGTACTAGGAAGAACCAATAACGCTATGCTCTGGGTCAACATGTTCTCTGGGGACTTTAAGGCGCTTGAGGAActgtctctt                     >  1:217014/15‑293 (MQ=255)`  
                `caatgaatacttggaggaagtcgaggaagtcgCACGACAGAAAGAAATTGACCGCGCTAAGGCCCGTAAAGAACGTCACGAGGGGCGCTTAGAGGCACGCAGATTCAAACGTCGCAACCGCAA‑GGCACGTAAAGCACACAAAGCTAAGCGCGAAAGAATGCTTGCTGCGTGGCGATGGGCTGAACGTCAAGAACGGCGTAACCATGAGGTAGCTGTAGATGTACTAGGAAGAACCAATAACGCTATGCTCTGGGTCAACATGTTCTCTGGGGACTTTAAGGCGCTTGAGGAACGAATcgcg                     >  1:525008/15‑301 (MQ=255)`  
                `caatgaatacttggaggaagtcgaggaagtcgCACGACAGAAAGAAATTGACCGCGCTAAGGCCCGTAAAGAACGTCACGAGGGGCGCTTAGAGGCACGCAGATTCAAACGTCGCAACCGCAA‑GGCACGTAAAGCACACAAAGCTAAGCGCGAAAGAATGCTTGCTGCGTGGCGATGGGCTGAACGTCAAGAACGGCGTAACCATGAGGTAGCTGTAGATGTACTAGGAAGAACCAATAACGCTATGCTCTGGGTCAACATGTTCTCTGGGGACTTTAAGGCGCTTGAGGAACGAATcgcc                     >  1:517651/15‑300 (MQ=255)`  
                `caatgaatacttggaggaagtcgaggaagtcgCACGACAGAAAGAAATTGACCGCGCTAAGGCCCGTAAAGAACGTCACGAGGGGCGCTTAGAGGCACGCAGATTCAAACGTCGCAACCGCAA‑GGCACGTAAAGCACACAAAGCTAAGCGCGAAAGAATGCTTGCTGCGTGGCGATGGGCTGAACGTCAAGAAAGGCGTAACCATGAGGTAGCTGTAGATGTACTAGGAAGAACCAATAACGCTATGCTCTGGGTCAACATGGTCTCAGGGGAATTTAAGGCGATTGAGGCActgtctctt                     >  1:647565/15‑293 (MQ=255)`  
                 `aatgaatacttggaggaagtcgaggaagtcgCACGACAGAAAGAAATTGACCGCGCTAAGGCCCGTAAAGAACGTCACTAGGGGCGCTTAGAGGCACGCAGATTCAAACGTCGCAACCGCAA‑GGCACGTAAAGCACACAAAGCTAAGCGCGAAAGAATGCTTGCTGCGTGGCGATGGGCTGAACGTCAAGAACGGCGTAACCATGAGGTAGCTGTAGATGTACTAGGAAGAACCAATAACGCTATGCTCTGGGTCAACATGTTCTCTGGGGACTTTAAGGCGCTTGAGGAACGAATCgcgc                    >  1:118450/14‑301 (MQ=255)`  
                  `atgaatacttggaggaagtcgaggaagtcgCACGACAGAAAGAAATTGACCGCGCTAAGGCCCGTAAAGAACGTCACGAGGGGCGCTTAGAGGCACGCAGATTCAAACGTCGCAACCGCAA‑GGCACGTAAAGCACACAAAGCTAAGCGCGAAAGAATGCTTGCTGCGTGGCGATGGGCTGAACGTCAAGAACGGCGTAACCATGAGGTAGCTGTAGATTTACTAGGAAGAACACATAACGCTATGCTCTGGGTCAACATGTTCTCTGGGGCCTTTAAGGAGCTTGAGGAACGAATcgcgag                   >  1:327408/13‑299 (MQ=255)`  
                  `atgaatacttggaggaagtcgaggaagtcgCACGACAGAAAGAAATTGACCGCGCTAAGGCCCGTAAAGAACGTCACGAGGGGCGCTTAGAGGCACGCAGATTCAAACGTCGCAACCGCAA‑GGCACGTAAAGCACACAAAGCTAAGCGCGAAAGAATGCTTGCTGCGTGGCGATGGGCTGAACGTCAAGAACGGCGTAACCATGAGGTAGCTGTAGATGTACTAGGAAGAACCAATAACGCTATGCTATGGGTCAACATGTTTTCTGGGGACTTTAAGGCGCTTAAGGGACGCATAgcgcc                   >  1:205890/13‑300 (MQ=255)`  
                  `atgaatacttggaggaagtcgaggaagtcgCACGACAGAAAGAAATTGACCGCGCTAAGGCCCGTAAAGAACGTCACGAGGGGCGCTTAGAGGAACGCAGATTCAAACGTCGCAACCGCAA‑GGCACGTAAAGCACACAAAGCTAAGCGCGAAAGAATGCTTGCTGCGTGGCGATGGGCTGAACGTCAAGAACGGCGTAACCATGAGGTAGCTGTAGATGTACTAGGAAGAACCAATAACGCTATGCTCTGGGTCAACATGTTCTCTGGGGACTTTAAGGCGCTTGAGGAACGAATCGCGCt                   >  1:153126/13‑301 (MQ=255)`  
                  `atgaatacttggaggaagtcgaggaagtcgCACGACAGAAAGAAATTGACCGCGCTAAGGCCCGTAAAGAAAGTCACGAGGGGCGCTTAGAGGCACGCAGATTCAAACGTCGCAACCGCAA‑GGCACGTAAAGCACACAAAGCTAAGCGCGAAAGAATGATTGCTGCGTGGCGATGGGCTGAACGTCAAGAACGGCGTAACCATGAGGTAGCTGTAGATGTGCTAGGAAGAACCAATAACGCTATGCTCTGGGTCAACATGTTATCTGGGGACTTTAAGGCGCTTGAGGAAAGAATCGCGCt                   >  1:66600/13‑301 (MQ=255)`  
                   `agaatacttggaggaagtcgaggaagtcgCACGACAGAAAGAAATTGACCGCGCTAAGGCCCGTAAAGAACGTCACGAGGGGCGCTTAGAGGCACGCAGATTCAAACGTCGCAACCGCAA‑GGCACGTAAAGCACACAAAGCTAAGCGCGAAAGAATGCTTGCTGCGTGGCGATGGGCTGAACGTCAAGAACGGCGTAACCATGAGGTAGCTGTAGATGTACTAGGAAGAACCAATAACGCTATGCTCTGGGTCAACATGTTCTCTGGGGACTTTAAGGCGCTTGATGAACGAATCGCGCTg                  <  1:252889/290‑1 (MQ=255)`  
                    `gaatacttggaggaagtcgaggaagtcgCACGACAGAAAGAAATTGACCGCGCTAAGGCCCGTAAAGAACGTCACGAGGGGCGCTTAGAGGCACGCAGATTCAAACGTCGCAACCGCAA‑GGCACGTAAAGCACACAAAGCTAAGCGCGAAAGAATGCTTGCTGCGTGGCGATGGGCTGAACGTCAAGAACGGCGTAACCATGAGGTAGCTGTAGATGTACTAGGAAGAACCAATAACGCTATGCTCTGGGTCCACATGTTCTCTGGGGACTTTAAGGCGCTTGAGGAACGAATCGCGCTGc                 >  1:183997/11‑301 (MQ=255)`  
                    `gaatacttggaggaagtcgaggaagtcgCACGACAGAAAGAAATTGACCGCGCTAAGGCCCGTAAAGAACGTCACGAGGGGCGCTTAGAGGCACGCAGATTCAAACGTCGCAACCGCAA‑GGCACGTAAAGCACACAAAGCTAAGCGCGAAAGAATGCTTGCTGCGTGGCGATGGGCTGAACGTCAAGAACGGCGTAACCATGAGGTAGCTGTAGATGTACTAGGAAGAACCAATAACGCTATGCTCTGGGTCAACATGTTCTCTGGGGACTTTAAGGCGCTTGAGGAACGACTCGCGCTGc                 >  1:567020/11‑301 (MQ=255)`  
                    `gaatacttggaggaagtcgaggaagtcgCACGACAGAAAGAAATTGACCGCGCTAAGGCCCGTAAAGAACGTCACGAGGGGCGCTTAGAGGCACGCAGATTCAAACGTCGCAACCGCAA‑GGCACGTAAAGCACACAAAACTAAGCGCGAAAGAATGCTTGCTGCGTGGCGATGGGCTGAACGTCAAGAACGGCGTAACCATGAGGTAGCTGTAGAGGTACTAGGAAGAACCAATAACGCGATGCTCTGGGTCAACATGTTCTCTGGGGACGTTAAGGCGCTTGAGGAACGAATctggctct                 >  1:327179/11‑294 (MQ=255)`  
                      `atataagaagcaggtcgaggaagtcaCACGACAGAAAGAAATTGACCGCGATAAGGCCCGTAAAGAACGTCACGAGTGGCGCTGAGAGGCACGCAGATGCAAACGTCGCAACCGCAA‑GGCACGTAAAGCACACAAAGCTAAGCGCGAAAGAATGCTGGCTGCGTGGCGATGGGCTGAACGTCAAGAACGGCGTAACCATGAGGTAGCTGTAGATGTACTAGGAAGAACCAATAACGCTATGCTCTGGGTCAACATGTTCTCTGGGGACTTTAAGGCGCTTGAGGAACGAATCGCGCTGCAc               <  1:465528/288‑1 (MQ=255)`  
                      `atacttgggggaagtcgaggaagtcgCACGACAGAAAGAAATTGACCGCGCTAAGGCCCGTAAAGAACGTCACGAGGGGCGCTTAGAGGCACGCAGATTCAAACGTCGCAACCGCAA‑GGCACGTAAAGCACACAAAGCTAAGCGCGAAAGAATGCTTGCTGCGTGGCGATGGGCTGAACGTCAAGAACGGCGTAACCATGAGGTAGCTGTAGATGTACTAGGAAGAACCAATAACGCTATGCTCTGGGTCAACATGTTCTCTGGGGACTTTAAGGCGCTTGAGGAACGAATCGCGCTGCAc               <  1:4907/292‑1 (MQ=255)`  
                      `atacttggaggaagtcgaggaagtcgCACGACAGAAAGAAATTGACCGCGCTAAGGCCCGTAAAGAACGTCACGAGGGGCGCTTAGAGGCACGCAGATTCAAACGTCGCAACCGCAA‑GGCACGTAAAGCACACAAAGCTAAGCGCGAAAGAATGCTTGCTGCGTGGCGATGGGCTGAACGTCAAGAACGGCGTAACCATGAGGTAGCTGTAGATGTACTAGGAAGAACCAATAACGCTATGCTCTGGGTCAACATGTTCTCTGGGGACTTTAAGGCGCTTGAGGAACGAATCGCGCTGCAc               <  1:594061/293‑1 (MQ=255)`  
                      `atacttggaggaagtcgaggaagtcgCACGACAGAAAGAAATTGACCGCGCTAAGGCCCGTAAAGAACGTCACGAGGGGCGCTTAGAGGCACGCAGATTCAAACGTCGCAACCGCAA‑GGCACGTAAAGCACACAAAGCTAAGCGCGAAAGAATGCTTGCTGCGTGGCGATGGGCTGAACGTCAAGAACGGCGTAACCATGAGGTAGCTGTAGATGTACTAGGAAGAACCAATAACGCTATGCTCTGGGTCAACATGTTCTCTGGGGACTTTAAGGCGCTTGAGGAACGAATCGCGCTGCAc               >  1:300021/9‑301 (MQ=255)`  
                      `atacttggaggaagtcgaggaagtcgCACGACAGAAAGAAATTGACCGCGCTAAGGCCCGTAAAGAACGTCACGAGGGGCGCTTAGAGGCACGCAGATTCAAACGTCGCAACCGCAA‑GGCACGTAAAGCACACAAAGCTAAGCGCGAAAGAATGCTTGCTGCGTGGCGATAGGCTGAACGTCAAGAACGGCGTAACCATGAGGTAGCTGTAGATGTACTAGGAAGAACCAATACCGCTATGCTCTGGGTCAACATGTTCTCTGGGGACTTTACGGCGCTTGAGGAACGAATCGCGCTTCAc               >  1:355361/9‑301 (MQ=255)`  
                       `tacttgggagaaggcgaggaagtcgCACGACAGAAAGAAATTGACCGCGCTAAGGCCCGTAAGGAACGTCACGAGGGGCCCTTCGAGCCACGCAGATTTAAACGTCGCAACCGACA‑GGCACGTAAAGCACACAAAGCTAAGCTCGAAAGAATGCTTGCTGCGTGGCTATGGGCTGAACGTCAAGAACGGCGTAACCATGATGTAGCTGTAGATGTACTAGGAAGAACCAATAACGCTATGCTCTTGGTCAACATGTTCTCTGGGGACTTTAAGGCGCTTGAGGAACGAATCGCGCTGCACt              <  1:143724/292‑1 (MQ=255)`  
                       `tacttggaggaagtcgaggaagtcgCACGACAGAAAGAAATTGACCGCGCTAAGGCCCGTAAAGAACGTCACGAGGGGCGCTTAGAGGCACGCAGATTCAAACGTCGCGACCGCAA‑GGCACGTAAAGCACACAAAGCTAAGCGCGAAAGAATGCTTGCTGCGTGGCGATGGGCTGAACGTCAAGAACGGCGTAACCATGAGGTAGCTGTAGATGTACTAGGAAGAACCAATAACGCTATGCTCTGGGTCAACATGTTCTCTGGGGACTTTAAGGCGCTTGAGGAACGAATCGCGCTGCAcc              >  1:508061/8‑300 (MQ=255)`  
                       `tacttggaggaagtcgaggaagtcgCACGACAGAAAGAAATTGACCGCGCTAAGGCCCGTAAAGAACGTCACGAGGGGCGCTTAGAGGCACGCAGATTCAAACGTCGCAACCGCAA‑GTCACGTAAAGCACACAAAGCTAAGCGCGAAAGAATGCTTGCTGCGTGGCGATGGGCTGAACGTCAAGAACGGCGTAACCATGAGGTAGCTGTAGATGTACTAGGACGAACCAATAACGCTATGCTCTGGGTCAACATGTTCTCTGGGGACTTTAAGGCGCTTGAGGAACGAATCGCGCTGCACt              >  1:602150/8‑301 (MQ=255)`  
                       `aacttggaggaagtcgaggaagtcgAACGACAGAAAGAAATTGACCGCGCTAAGGCCCGTAAAGAACGTCACGAGGGGCGCTTAGAGGCACGCAGATTCAAACGTCGCAACCGAAA‑GGCACGTAAAGCACACAAAGCTAAGCGCGAAAGAATGCTTGCTGCGTGGCGATGGGCTGAACGTCAAGAACGGCGTAACCATGAGGTAGCTGTAGATGTACTAGGAAGAACCAATAACGCTATGCTCTGGGTCAACATGTTCTCTGGGGACTTTAAGGCGCTTGAGGAACGAATCGCGCTGCACt              <  1:339926/294‑1 (MQ=255)`  
                        `acttggaggaggtcgaggaagtcgAGCGACAGAAAGGAATTGACCGCGGTAAGGCCCGTAAAGAACGTCACGAGGGGCGCTTGGAGGCACGCAGATTCAAACGGCGCAACCGCAA‑GGCACGTAAAGCACACAAAGCTAAGCGCGAAAGAATGCGTGCTGCGTGGCGATGGGGTGAACGTCAAGAACGGCGTAACCATGAGGTAGCTGTAGATGTACTCGGAAGAACCAATAACGCTATGCTCTGGGTCAACATGTTCTCTGGGGACTTTAAGGCGCTTGAGGAACGAATCGCGCTGCACTg             >  1:605036/7‑301 (MQ=255)`  
                        `acttggaggaagtcgaggaagtcgCACGACAGAAAGAAATTGACCGCGCTAAGGCCCTTAAAGAACGTCACGAGGGGCCCTTAAAGGCACGCAGATTCAAACGTCGAAACCGCAA‑GGCACGTAAAGCACACAAACCTAAGCGCGAAGAAATGCTTGCTCCGTGGCGATGGGCTGAACGTCAAGAACGGCGTAACCATGAGGAAGCTGTAAATGAACTAGGAAGAACCAAAAACCCCATACTCTGGTTCAACATGTTCTCTGGGGACTTTAAGGCGCTGCAGGAACGAATCGCGCTGCACTg             >  1:315973/7‑301 (MQ=255)`  
                        `acttggaggaagtcgaggaagtcgCACGACAGAAAGAAATTGACCGCGCTAAGGCCCGTAAAGAACGTCACGAGGGGCGCTTAGAGGCACGCAGATTCAAACGTCGCAACCGCAA‑GGCACGTAAAGCACACAAAGCTAAGCGCGAAACAATGCTTGCTGCGTGGCGATGGGCTGAACGTCAAGAACGGCGTAACCATGAGGTAGCTGTAGATGTACTAGGAAGAACCAATAACGCTATGCTCTGGGTCAACATGTTCTCTGGGGACGTTAAGGCGCTTGAGGAACGAATCGCGCTGCACTg             >  1:650083/7‑301 (MQ=255)`  
                         `tgtggaggaagtcgaggaaggcgAACGACAGAAAGAAATTGACGGAGCGAAGGCCCGAAAAGGACGTCAGGAAGGGCGCTTAGAGGCACGCAGATTCAAACGTCGCAACCGCAA‑GGCACGGAAAGCACACAAATCTAAGCGCGAAAGGATGCTTGCTGCGTGGCGATGGGCTGAACGTCAAGAACGGCGTAACCATGAGGTAGCTGTAGATGTACTAGGAAGAACCAATAACGCTATGCTCTGGGTCAACGTGTTCTCTGGGGACTTTAAGGCGCTTGAGGAACGAATCGCGCTGCACTgg            <  1:421781/296‑1 (MQ=255)`  
                         `cttggaggaagtcgaggaagtcgCACGACAGAAAGAAATTGACCGCGCTAAGGCCCGTAAAGAACGTCACGAGGGGCGCTTAGAGGCACGCAGATTCAAACGTCGCAACCGCAA‑GGCACGTAAAGCACACAAAGCTACGCGCGAAAGAATGCTTGCTGCGTGGCGATGGGCTGAACGTCAAGAACGGCGTAACCATGAGGTAGCTGTAGATGTACTAGGGAGAACCAATAACGCTATGCTCTGGGTCAACATGTTCTCTGGGGACTTTAAGGCGCTTGAGGAACGAATCGCGCTGCACTgg            >  1:294398/6‑301 (MQ=255)`  
                         `cttggaggaagtcgaggaagtcgCACGACAGAAAGAAATTGACCGCGCTAAGGCCCGTAAAGAACGTCACGAGGGGCGCTTAGAGGCACGCAGATTCAAACGTCGCAACCGCAA‑GGCACGTAAAGCACACAAAGCTAAGCGCGCAAGAATGCTTGCTGCGTGGCGATGGGCTGAACGTCAAGAACGGCGTAACCATGAGGTAGCTGTAGATGTACTAGGAAGAACCAATAACGCTATGCCCTGGGTCAACATGTTCTCTGGGGACTTTAAGGCGCTTGAGGAACGAATCGCGCTGCACTgg            >  1:666776/6‑301 (MQ=255)`  
                         `cttggaggaagtcgaggaagtcgCACGACAGAAAGAAATTGACCGCGCTAAGGCCCGTAAAGAACGTCACGAGGGGCGCTTAGAGGCACGCAGATTCAAACGTCGCAACCGCAA‑GGCACGTAAAGCACACAAAGCTAAGCGCGAAAGAATGCTTGCTGCGTGGCGATGGGCTGAACGTCAAGAACGGCGTAACCATGAGGTAGCTGTAGATGTACTAGGAAGAACCAATAACGCTATGCTCTGGGTCAACATGTTCTCTGGGGACTTTAAGGCGCTTGAGGAACGAATCGCGCTGCACTgg            >  1:570027/6‑301 (MQ=255)`  
                         `cttggaggaagtcgaggaagtcgCACGACAGAAAGAAATTGACCGCGCTAAGGCCCGTAAAGAACGTCACGAGGGGCGCTTAGAGGCACGCAGATTCAAACGTCGCAACCGCAA‑GGCACGTAAAGCACACAAAGCTAAGCGCGAAAGAATGCTTGCTGCGTGGCGATGGGCTGAACGTCAAGAACGGCGTAACCATGAGGTAGCTGTAGATGTACTAGGAAGAACCAATAACGCTATGCTCTGGGTCAACATGGTCTCTGGGGACTTTAAGGCGCTTGAGGAACGAATCGTGCTGCACTgg            >  1:214567/6‑301 (MQ=255)`  
                         `cttggaggaagtcgaggaagtcgAACGACAGAAAGAAATTGACCGCGCTAAGGCCCGTAAAGAACGTCACGAGGGGCGATTAGAGGCACGCAGATTCAAACGTCGCAACCGCAA‑GGCACGTAAAGCACACAAAGCTAAGCGCGAAAGAATGCTTGCTGCGTGGCGATGGGCTGAACGTCAAGAACGGCGTAACCATGAGGTAGCTGTAGTTGTACTAGGAAGAACCACTAACGCTATGCTCTGGGTCAACATGTTCTCTGGGGACTTTAAGGCGCTTGAGGAACGAATCGCGCTGCACTgg            >  1:113495/6‑301 (MQ=255)`  
                         `caatgaatacttggaggaagtagCAAGACAGAAAGAAATTGAACGCGCAAAGGCCCGTACAGAATGTCACGAGAGGCGCTTAGAGGAACGCAGATTCAAACGTCGCAACCGAAA‑GGCACGTAAAGCACACAAAGCTAAGCGCGAAAGACTGCTTGCTGCGTGGCGATGGGCTGAAAGACACGAACGGCGTAACCATGAGGTAGATGTAGCAGGACTATGAAGAACAAATAACGCTATCCTCTGGTCCACCATGTTACCTGGGGACTCAGAGGTGCTAGAGGACCGTATAGAGCTGCAcctg            >  1:647318/14‑298 (MQ=255)`  
                         `aagggaggaagtcgaggaagtcgCACGACAGAAAGAAATTGACCGCGCTAAGGCCCGTAAAGAACGTCACGAGGGGCGCTTAGAGGCACGCAGATTCAAACGTCGCAACCGCAA‑GGCACGTAAAGCACACAAAGCTAAGCGCGAAAGAATGCTTGCTGCGTGGCGATGGGCTGAACGTCAAGAACGTCGTAACCATGAGGTATCTGTAGATGTACTAGGAAGAACCAATAACGCTATGCTCTGGGTCAACATGTTCTCTGGGGACTTTAAGGCGCTTGAGGAACGAATCGCGCTGCACTgg            <  1:573058/296‑1 (MQ=255)`  
                           `tggaggaagtcgaggaagtagCAAGACAGAAAGAAATTGACCGCGCTAAGGCCCGTAAAGAACGTCACGAGGGGCTCTTAGAGGAACGCAGATTCAAACGTCGCAACCGCAA‑GGCACGTAAATCACACAAAGCTAAGCGCTAAAGAATGCTTGCTGCGTGGCGATGGGCTGAACGTCAAGAACGGCGTAACCATGAGGTAGCTGTAGATGTACTAGGAAGAACCAATAACGCTATGCTCTGGGTCAACATGTTCTCTGGGGACTTTAAGGCGCTTGAGGAACGAATCGCGCTGCACTGGCg          <  1:315111/298‑1 (MQ=255)`  
                           `tggaggaaggcgaggaagtcaCACGACAGAAAGAAATTGACCGCGCTAAGGCCCGTAAAGAACGGCACGAGGGGCGCTTAGAGGCACGCAGATTCAAACGTCGCAACCGCAA‑GGCACGTAAAGCACACAAATCTAAGCGCGAAAGAATGCTTGCTGCGTGGCGATGGGCTGAACGTAAAGAACGGCGTAACCATTAGGTAGCTGTAGAGGTACTAGGAAGAACCAATAACGCTATGCTCTTGGTCAACATGTTCTCTGGGGACTTTAAGGCGCTTGAGGAACGAATCGCGCTGCACTGGCg          <  1:71814/298‑1 (MQ=255)`  
                           `gggaggaagtcgaggaagtcgCACGACAGAAAGAAATTGACCGCGCTAAGGCCCGTAAAGAACGTCACGAGGGGCGCTTAGAGGCACGCAGATTCAAACGTCGCAACCGCAA‑GGCACGTAAAGCACACAAAGCTAAGCGCGAAAGAATGCTTGCTGCGTGGCGATGGGCTGAACGTCAAGAACGGCGTAACCATGAGGTAGCTGTAGATGTACTAGGAAGAACCAATAACGCTATGCTCTGGGTCAACATGTTCTCTGGGGACTTTAAGGCGCTTGAGGAACGAATCGCGCTGCACTGGCg          <  1:345636/298‑1 (MQ=255)`  
                           `aggaggaagtcgaggaagtcgCACGACAGAAAGAAATTGACCGCGCTAAGGCCCGGAAAGAACGTCACGAGGGGCGCTTAGAGGCACGCAGATTCAAACGTCGCAACCGCAA‑GGCACGTAAAGCACACAAAGCTAAGCGCGAAAGAATGCTGTCTGCGTGGCGATGGGCTGAACTTCAAGAACGGCGTAACCATGAGGTAGCTGTAGATGTACTAGGAAGAACCAATAACGCTATGCTCTGGGTCAACATGTTCTCTGGGGACTTTAATGCGCTTGAGGAACGAATCGCGCTGCACTGGCg          <  1:643654/298‑1 (MQ=255)`  
                            `ggaggaagtcgaggaagtcgCACGACAGAAAGAAATTGACCGCGCTAAGGCCCGTAAAGAACGTCACGAGGGGCGCTTAGAGGCACGCAGATTCAAACGTCGCAACCGCAA‑GGCACGTAAAGCACACAAAGCTAAGCGCGAAAGAATGCTTGCTGCGTGGCGATGGGCTGAACGTCAAGAACGGCGTAACCATGAGGTAGCTGTAGATGTACTAGGAAGAACCAATAACGCTATGCTCTGGGTCAACATGTTCTCTGGGGACTTTAAGGCGCTTGAGGAACGAATCGCGCTGCACTGGCGt         >  1:271446/3‑301 (MQ=255)`  
                            `ggaggaagtcgaggaagtcgCAAGACAGAAAGAAATTGACCGCGCTAAGGCCCGTAAAGAACGTCACGAGGGGCGCTTAGAGGCACGCAGATTCAAACGTCGCAACCGCAA‑GGCACGTAAAGCACACAAAGCTAAGCGCGAAAGAATGCTTGCTGAGTGGCGATGGGCTGAACGTCAAGAACGGCGTAACCATGAGGTAGCTGTAGATGTACTAGGAGGAACCAATAACGCTATGCTCTGGGTCAACATGTTCTCTGGGGACTTTAAGGCGCTTGAGGAACGAATCGCGCTGCACTGGCGt         <  1:146443/299‑1 (MQ=255)`  
                            `gaaGGAAGTCGAGGAAGTCGCAGGACAGAAAGAAATTTACCGGGCGAAGGCCCTTAAAGAATGTCACAAGGGGCGCTTAGAGGAACGCAGATTCAAACGTCGCAACCGCAA‑GGCACGTAAAGCACACAAAGCTAAGCGCGAAAGAATGCTTGCTGCGTGGCGATGGGCTGAACGTCAAGAACGGCGTAACCATGAGGTAGCTGTAGATGTACTAGGAAGAACCAATAACGCTATGCTCTGGGTCAACATGTTCTCTGGGGACTTTAAGGCGCTTGAGGAACGAATCGCGCTGCACTGGCGt         <  1:48161/300‑1 (MQ=255)`  
                             `gaggaagtcgaggaagtcgCACGACAGAAAGAAATTGACCGCGCTAAGGCCCGTAAAGAACGTCACGAGGGGCGCTTAGAGGCACGCAGATTCAAACGTCGCAACCGCAA‑GGCACGTAAAGCACACAAAGCTAAGCGCGAAAGAATGCTTGCTGCGTGGCGATGGGCTGAACGTCAAGAACGGCGTAACCATGAGGTAGCTGTAGATTTACTAGGAAGAACCAATAACACTATTATCTGGGGCAACATGTTCTCTGTGGACTTTAAGGCGCCTGAGGAACGAATCGCACTGCACTTGCTTa        >  1:495108/2‑301 (MQ=255)`  
                             `gaggaagtcgaggaagtcgCACGACAGAAAGAAATTGACCGCGCTAAGGCCCGTAAAGAACGTCACGAGGGGCGCTTAGAGGCACGCAGATTCAAACGTCGCAACCGCAA‑GGCACGTAAAGCACACAAAGCTAAGCGCGAAAGAATGCTTGCTGCGTGGCGATGGGCTGAACGTCAAGAACGGCGTAACCATGAGGTAGCTGTAGATGTACTAGGAAGAACCAATAACGCTATGCTCTGGGTCAACATGTTCTCTGGGGACTTTAAGGCGCTTGAGGAACGAATCTCGCTGCACTGGCGTa        >  1:504695/2‑301 (MQ=255)`  
                             `gaggaagtcgaggaagtcgCACGACAGAAAGAAATTGACCGCGCTAAGGCCCGTAAAGAACGTCACGAGGGGCGCTTAGAGGCACGCAGATTCAAACGTCGCAACCGCAA‑GGCACGTAAAGCACACAAAGCTAAGCGCGAAAGAATGCTTGCTGCGTGGCGATGGGCTGAACGTCAAGAACGGCGTAACCATGAGGTAGCTGTAGATGTACTAGGAAGAACCAATAACGCTATGCTCTGGGTCAACATGTTCTCTGGGGACTTTAAGGCGCTTGAGGAACGAATCGCGCTGCACTGGCGTa        >  1:495107/2‑301 (MQ=255)`  
                                `gaagtcgaggaagtcgCACGACAGAAAGAAATTGACCGCGCTAAGGCCCGTAAAGAACGTCACGAGGGGCGCTTAGAGGCACGCAGATTCAAACGTCGCAACCGCAT‑GGCACGTAAAGCACACAAAGCTAAGCGCGAAAGAATGCTTGCTGCGTGGCGATGGGCTGAACGTCAAGAACGGCATAACCATGAGGTAGCTGTAGATGTACTAGGAAGAACCAATAACGCTATGCTCTGGGTCAACATGTTCTCTGGGGACTTTAAGGCGCTTGAGGAACGAATCGCGCTGCACTGGCgctggt     >  1:196704/1‑296 (MQ=255)`  
                                `gaagtcgaggaagtcgCACGACAGAAAGAAATTGACCGCGCTAAGGCCCGTAAAGAACGTCACGA‑GGGCGCTTAGAGGCACGCAGATTCAAACGTCGCAACCGCAA‑GGCACGTAAAGCACACAAAGCTAAGCGCGAAAGAATGCTTGCTGCGTGGCGATGGGCTGAACGTCAAGAACGGCGTAACCATGAGGTAGCTGTAGATGTACTAGGAAGAACCAATAACGCTATGCTCTGGGTCAACATGTTCTCTGGGGACTTTAAGGCGCTTGAGGAACGAATCGCGCTGCACTGGCGTAATGc    <  1:101714/301‑1 (MQ=255)`  
                                `gaagtcgaggaagtcgCAAGACAGAAAGAAAGTGACCGCGCTAAGGCCCGTAAAGAACGTCACGAGGGGCGCTTAGAGGCACGCAGATTCAAACGTCGCAACCGCAA‑GGCACGTAAAGCACACAAAGCTAAGCGCGAAAGAATGCTTGTTGCGTGGCGATGGGCTGAACGTCAAGAACGGCGTAACCATGAGGTAGCTGTAGATGTACTAGGAAGAACCAATACCGCTATGCTCTGGGTCAACATGTTCTCTGGGGTCTTTAAGGCGCTTGAGGAACGAATCGCGCTGCACTGGCGTAATg     <  1:95301/301‑1 (MQ=255)`  
                                `gaagtcgagaaagtcgAACGACAAAAAGAAATTACCCGCGCAAGGGCCCGAAAAGAACTCAACGGGGGCGGCTAAGAGGCACGAAGATTC‑AACGGCCCAACCGGAAGGGCACGAAAGCCACCCAAGCGTAAGCGCGAAGGAAGCCTTCCTGCGTGGGGATGGCCTGAAGGTAAGGACCGCCGTAACCAGGAGTTGGCGGTAGATTTCCTAGGAAAACCCATTAACGCTATGCTC‑GGGTCAACATGTTCTCTGGGGACTTTAAGGCGCTTGAGGAACGAATCGCGCTGCACTGGCGTAATGc    <  1:535688/301‑1 (MQ=255)`  
                                 `aggtcgaggaagtcgCACGACAGAAAGAAATTGACCGCGCTAAGGCCCGTAAAGAACGTCACGAGGGGCGCTTAGAGGCACGCAGATTCAAACGTCGCAACCGCAA‑GGCACGTAAAGCACACAAAGCTAAGCGCGAAAGAATGCTTGCTGCGTGGCGATGGGCTGAACGTCAAGAACGGCGTAACCATGAGGTAGCTGTAGATGTACTAGGAAGAACCAATAACGCTATGCTCTGGGTCAACATGTTCTCTGGGGACTTTAAGGCGCTTGAGGAACGAATCGCGCTGCACTGGCGTAATGc    <  1:585218/299‑1 (MQ=255)`  
                                 `acttgagggaagtcgCACGACAGAAAGAACTTCACCTCGCTAAGTCCCGTAAATAACGTCACGAGGGGCGCTTCGAGGCACGCAGATTCAAACGTCGCAACCGCAA‑GGCACGTAAAGCACACCAAGCTAAGCGCGAAAGAATGCTTGCTGCGTGGCGATGGGCTGAACGTCAAGAACGGCGTAACCATGAGGTAGCTGTAGATGTACTAGGAAGAACCAATAACGCTATGCTCTGGTTCAACATGTTCTCTGGGGACTTTAAGGCGCTTGAGGAACGAATCGCGCTGCACTGGCGTAATGc    <  1:55375/294‑1 (MQ=255)`  
                                 `aagtcgaggaagtcgCACGACAGAAAGAAATTGACCGCGCTAAGGCCCGTAAAGAACGTCACGAGGGGCGCTTAGAGGCACGCAGATTCAAACGTCGCAACCGCAA‑GGCACGTAAAGCACACAAAGCTAAGCGCGAAAGAATGCTTGCTGCGTGGCGATGGGCTGAACGTCAAGAACGGCGTAACCATGAGGTAGCTGTAGATGTACTAGGAAGAACCAATAACGCTATGCTCTGGGTCAACATGTTCTCTGGGGACTTTAAGGCGCTGGGGGAACGAATCGCGCTGCACTGGCGTAATGc    >  1:86045/1‑301 (MQ=255)`  
                                 `aagtcgaggaagtcgCACGACAGAAAGAAATTGACCGCGCTAAGGCACGTAAAGAACGTCACGAGGGGCGCTTAGAGGCACGCAGATTCAAACGTCGCAACCGCAA‑GGCACGTAAAGCACACAAAGCTAAGCGCGAAAGAATGCTTGCTGCGTGGCGATGGGCTGATCGTCAAGAACGGCGTAACCATGAGGTAGCTGTAGATGTACTAGGAAGAACCAATAACGCTATGCTCTGGGTCAACATGTTCTCTGGGGACTTTAAGGCGCTTGAAGAACGAATCGCGCTGCACTGGCGTAATGc    <  1:209559/301‑1 (MQ=255)`  
                                 `aagtcgaggaagtcgCACGAAAGAAAGAAATTGACCGCGCTAAGGCCCGTAAAGAACGTCACGAGGGGCGCTTAGAGGCACGCAGATTCAAACGTCGCAACCGCAA‑GGCACGTAAAGCACACAAAGCTAAGCGCGAAAGAATGCTTGCTGCGTGGCGATGGGCTGATCGTCAAGAACGGCGTAACCATGAGGTAGCTGTAGATGTACTAGGAAGAACCAATAACGCTATGCTCTGGGTCAACATGTTCTCTGGGGACTTTAAGGCGCTTGAAGAACGAATCGCGCTGCACTGGCGTAATGc    <  1:209539/301‑1 (MQ=255)`  
                                 `aagtcgaggaagtcgAACGACAGAAAGAAATTGACCGCGCTAAGGCCCGTAAAGAACGTCACGAGGGGCGCTTAGAGGCACGCAGATTCAAACGTCGCAACCGCAA‑GGCACGTAAAGCACACAAAGCTAAGCGCGAAAGAATGCTTGCTGCGTGGCGATGGGCTGAACGTCAAGAACGGCGTAACCATGAGGTAGCTGTAGATGTACTAGGAAGAACCAATAACGCTATGCTCTGGGTCAACATGTTCTCTGGGGACTTTAAGGCGCTTGAGGAACGAATCGCGCTGCACTGGCGTAATGc    <  1:263705/301‑1 (MQ=255)`  
                                 `aaggcgaggaagtcgCAAGACAGAAAGAAATTGACCGCGCGAAGGCCAGTAAAGAACGTCACGGTGTGCGCGTAGAGGAACGCAGATTCAAACGGCGCAACCGAAA‑GGCACGTAAAGCACACAAAGCTAAGCGCGAAAGAATGCTTGCTGCGTGGCGATGGGCTGAACGTCAAGAACTGCGTAACCATGAGGTAGCTGTAGATGTACTAGGAAGAACCAATAACGCTATGCTCTGGGTCAACATGTTCTCTGGGGACTTTAAGGCGCTTGAGGAACGAATCGCGCTGCACTGGCGTAAtcc    <  1:614328/301‑3 (MQ=255)`  
                                 `aagacgaggaagtcgAACGACAGAAAGAAATGGACCGAGGTAAGGCCAGTAAAGAACGGCACGAGGGGCGCTTAGAAGAACGCAGATTCAAACGTCGCAACCGCAA‑GGAACGTAAAGCACACAAAGCTAAGCGCGAAAGAATGCTTGCTGCGTGGCGATGGGCTGAACGTCAAGAACGGCGTAACCATGAGGTAGCTGTAGATGTACGAGGAAGAACCAATAACGCTATGCTCTGGGTCAACATGTTCTCTTGGGACTTTAAGGCGCTTGAGGAACGAATCGCGCTGCACTGGCGTAATGc    <  1:65126/301‑1 (MQ=255)`  
                                   `gtcgaggaagtcgCACGACAGAAAGAAATTGACCGCGCTAAGGCCCGTAAAGAACGTCACGAGGGGCGCTTAGAGGCACGCAGATTCAAACGTCGCAACCGCAA‑GGCACGTAAAGCACACAAAGCTAAGCGCGAAAGGATGCTTGCTGCGTGGCGATGGGCTGAACGTCAAGAACGGCGTAACCATGAGGTAGCTGTAGATGTACTAGGAAGAACCAATAACGCTATGCTCTGGGTCAACATGTTCTCTGGGGACTTTAAGGCGCTTGAGGAACGAATCGCGCTGCACTGGCGTAATGCtt  >  1:285545/1‑300 (MQ=255)`  
                                   `gtcgaggaagtcgCACGACAGAAAGAAATTGACCGCGCTAAGGCCCGTAAAGAACGTCACGAGGGGCGCTTAGAGGCACGCAGATTCAAACGTCGCAACCGCAA‑GGCACGTAAAGCACACAAAGCTAAGCGCGAAAGAATGCTTGCTGCGTGGCGATGGGCTGAACGTCAAGAACGGCGTAACCATGAGGTAGCTGTAGATGTACTAGGAAGAACCAATAACGCTATGCTCTGGGTCAACATGTTCTCTGGGGACTTTAAGGCGCTTGAGGAACGAATCGCGCTGCACtctgtctcttata  >  1:344796/1‑289 (MQ=255)`  
                                   `gtcgaggaagtcgCACGACAGAAAGAAATTGACCGCGCTAAGGCCCGTAAAGAACGTCACGAGGGGCGCTTAGAGGCACGCAGATTCAAACGTCGCAACCGCAA‑GGCACGTAAAGCACACAAAGCTAAGCGCGAAAGAATGCTTGCTGCGTGGCGATGGGCTGAACGTCAAGAACGGCGTAACCATGAGGTAGCTGTAGATGTACTAGGAAGAACCAATAACGCTATGCCCTGGGTCAACATGTTCTCTGGGGACTTTAAGGCGCTTGAGGAACGAATCGCGCTGCACTGGCGTAATGCtt  >  1:604527/1‑300 (MQ=255)`  

                                           `|`                                                                                                                                                                                                                                                                                                       
`CATTCAGCATCGACCCTGAGGAACTCATCAAGGAAGTCGAGGAAGTCGCACGACAGAAAGAAATTGACCGCGCTAAGGCCCGTAAAGAACGTCACGAGGGGCGCTTAGAGGCACGCAGATTCAAACGTCGCAACCGCAA‑GGCACGTAAAGCACACAAAGCTAAGCGCGAAAGAATGCTTGCTGCGTGGCGATGGGCTGAACGTCAAGAACGGCGTAACCATGAGGTAGCTGTAGATGTACTAGGAAGAACCAATAACGCTATGCTCTGGGTCAACATGTTCTCTGGGGACTTTAAGGCGCTTGAGGAACGAATCGCGCTGCACTGGCGTAATGCTG  >  NC_001604.1/2694‑3029`

  

Alignment Legend

Aligned base mismatch/match (shaded by quality score): `ATCG/ATCG` `< 3 ≤` `ATCG/ATCG` `< 9 ≤` `ATCG/ATCG` `< 17 ≤` `ATCG/ATCG` `< 33 ≤` `ATCG/ATCG` `< 38 ≤` `ATCG/ATCG`

Unaligned base: `atcg`    Masked matching base: `atcg`    Alignment gap: `‑`    Deleted base: `‑`

Reads not counted as support for junction

read\_name Not counted due to insufficient overlap past the breakpoint.

read\_name Not counted due to not crossing MOB target site duplication.
